# Multimodal Rehabilitation in Spinal Cord Lesion: Comparative Outcomes of Vojta Therapy and Lokomat Training

**DOI:** 10.3390/medicina61112041

**Published:** 2025-11-15

**Authors:** Anamaria Gherle, Carmen Delia Nistor-Cseppento, Liviu Lazar, Ștefania Deac, Mirela Elena Bodea, Florin Mihai Marcu, Sebastian Tirla, Mariana Lidia Cevei

**Affiliations:** 1 Doctoral School of Biomedical Sciences, Faculty of Medicine and Pharmacy, University of Oradea, 410087 Oradea, Romania; amgherle@uoradea.ro (A.G.); stefaniacristea95@yahoo.ro (Ș.D.); mirelabodea16@gmail.com (M.E.B.); tirlasebastian@gmail.com (S.T.); 2 Department of Psycho-Neurosciences and Recovery, Faculty of Medicine and Pharmacy, University of Oradea, 410087 Oradea, Romania; fmarcu@uoradea.ro (F.M.M.); cevei_mariana@uoradea.ro (M.L.C.)

**Keywords:** spinal cord lesion, robotic gait training (Lokomat^®^), Vojta Therapy, Modified Ashworth Scale, quality of life

## Abstract

*Background and Objectives*: Spinal cord lesion is a severe disorder of the central nervous system, leading to partial or complete interruption of nerve impulse transmission between the brain and the periphery and causing severe neurological and functional deficits. Conventional rehabilitation offers limited outcomes, while robotic gait training (Lokomat^®^) and Vojta Therapy have shown benefits individually. Evidence on their combined effect remains scarce. To evaluate the combined effect of Vojta Therapy and Lokomat-assisted gait training on motor recovery, functional independence, and quality of life in SCL patients. *Materials and Methods*: A retrospective clinical study was conducted on 205 patients with traumatic and non-traumatic SCL. Patients were allocated to four groups: (F)—conventional rehabilitation; (V)—conventional + Vojta; (L)—conventional + Lokomat; (VL)—conventional + Vojta + Lokomat. Assessments included the ASIA Impairment Scale (AIS), ASIA motor/sensory scores, spasticity (Modified Ashworth Scale, MAS), functional independence (Functional Independence Measure, FIM), and health-related quality of life (EQ-5D), performed at admission and discharge. Statistical analyses comprised paired *t*-tests, Wilcoxon signed-rank tests, chi-square tests, Kruskal–Wallis with Dunn’s post hoc corrections, and linear regression. *Results*: The most frequent lesion levels were C7 (21%) and L1 (20%). All groups showed improvement in FIM scores, with the greatest gains in the VL group (from 79.25 to 84.79, *p* < 0.05). Post hoc analysis confirmed significantly higher FIM outcomes in VL compared with L. Regression analysis identified the ASIA motor score as the strongest predictor of functional independence (β = 0.76, *p* < 0.001), with VL group membership adding +10.3 points (*p* = 0.004). EQ-5D indicated persistent deficits in mobility and self-care, especially in VL patients, consistent with higher lesion severity. *Conclusions*: Combining Vojta Therapy with Lokomat training provides additional functional benefits compared with Lokomat or Vojta alone. Multimodal individualized rehabilitation appears promising for patients with spinal cord lesions. Prospective randomized controlled trials with long-term follow-up are warranted.

## 1. Introduction

Traumatic spinal cord injuries (TSCIs) represent one of the most severe forms of trauma, with a major impact on patients’ functionality and quality of life [1]. Spinal cord lesions result in complex, often devastating neurological deficits due to partial or complete impairment of motor, sensory, and autonomic functions below the level of injury [2]. Clinically, the severity and neurological level of the lesion allow classification into several syndromes: tetraplegia/tetraparesis and paraplegia/paraparesis [3,4]. These neurological deficits considerably reduce functional independence and patients’ quality of life.

Rehabilitation strategies, including overground gait training and body-weight-supported treadmill training (BWSTT), have aimed at stimulating neuroplasticity, task-specific training, and compensatory techniques, with the goal of promoting motor recovery and improving gait performance [5,6]. Robotic systems may include treadmill-based platforms, partial body weight support (BWS) devices, and exoskeletons that guide the lower limbs through successive phases of stepping—an example being Lokomat^®^. In recent years, the integration of Lokomat^®^ into neurorehabilitation protocols has shown promising results in optimizing motor control of the lower limbs, stimulating neuroplasticity, and improving gait parameters, in both complete and incomplete spinal cord lesions [7].

Training with Lokomat provides intensive, repetitive, task-specific locomotor activity in a safe and controlled environment, becoming a central element of robotic rehabilitation [7,8].

In parallel, Vojta Therapy has gained interest in neurological rehabilitation. Vojta Therapy activates reflex locomotor patterns by applying pressure on specific body zones, aiming to stimulate global motor responses and improve postural control, even in cases with severe motor deficits [9]; it stimulates the brain’s neural networks, promoting the activation and reorganization of neural circuits [10]. Vojta Therapy is effective in disorders of the central nervous system, but it has not been well studied in TSCI. Recent research has shown that Vojta stimulation can increase muscle activation and cerebral perfusion. However, there are few rigorous studies evaluating the effectiveness of Vojta therapy in TSCI [10,11,12,13]. This therapy enhances the benefits of robotic therapy, such as Lokomat, by increasing training efficiency, improving motor control, balance, and coordination. In combination, these approaches can lead to superior results compared to conventional therapies, as Vojta therapy optimizes neuroplastic response, facilitating adaptation and consolidation of motor functions. In conclusion, integrating Vojta therapies with robotic interventions is a promising strategy for enhancing functional recovery, allowing for more intensive, repetitive, and personalized treatment with longer-lasting potential and a positive impact on clinical outcomes [14].

In essence, the rationale for this study was that combining these two methods would enhance the functional outcome for the patients with medular lesions, compared with standard rehabilitation programs alone, based on the hypothesis that pregait Vojta priming enhances Lokomat training effects by improving proximal stability and recruiting deep trunk stabilizers and postural chains.

By evaluating clinical parameters such as spasticity, functional independence, gait capacity, and mobility, this study seeks to contribute to the optimization of individualized rehabilitation protocols that could include Vojta therapy in combination with Lokomat training for patients with spinal cord lesions [15]. A secondary objective was the evaluation of predictive factors for the FIM independence score. The gap of the study is the lack of a consistent RCT directly testing Vojta therapy versus Lokomat alone and versus conventional therapy, so the synergistic effect remains unproven.

## 2. Materials and Methods

### 2.1. Design of the Study

A retrospective observational clinical study was conducted at the “Băile Felix Medical Rehabilitation Clinical Hospital”, including patients admitted between November 2022 and December 2023 for spinal-cord-injury-related tetraplegia/tetraparesis or paraplegia/paraparesis of traumatic or non-traumatic origin. The study design was retrospective, relying on clinical and functional data recorded during hospitalization. Patients were assessed at admission and at discharge. The study was approved by the local Ethics Committee of the Hospital (no. of approval: 2435/23 February 2023) and was carried out in accordance with the principles of the Declaration of Helsinki. All participants provided written informed consent prior to their inclusion in the study.

### 2.2. Inclusion/Exclusion Criteria

Inclusion criteria were patients with a confirmed diagnosis of spinal cord lesion of either traumatic or non-traumatic etiology (degenerative cervical and lumbar myelopathy), operated disc herniations with secondary myelopathy, operated intramedulary tumors, operated congenital arterio-venous malformations, interruption of spinal cord blood flow such as operated spinal cord fistulas, transverse myelitis, operated syringomyelia, presenting with tetraplegia or paraplegia, aged between 18 and 85 years old, and with a disease onset of at least one month prior to inclusion. Exclusion criteria were age below 18 or above 85 years; hemodynamic instability, unstable fractures, severe cognitive impairment, severe comorbidities contraindicating therapy, patients with tetraparesis/paraparesis due to other etiologies (such as multiple sclerosis, amyotrophic lateral sclerosis, bipyramidal syndrome following brain lesions; viral encephalitis; cerebral palsy; chronic inflammatory demyelinating polyneuropathy; diabetic polyneuropathy, traumatic brain injury); and patients in whom standardized assessments could not be performed.

### 2.3. Study Tools

All study participants were assessed using the following evaluation scales:

➢ American Spinal Injury Association (ASIA) Scale—the international standard for the neurological assessment of patients with spinal cord injury [2].
➢ ASIA Impairment Scale (AIS)- an integral part of the International Standards for Neurological Classification of SCI (ISNCSCI). The AIS grades the severity of spinal cord injury (Table 1) [16].
➢ Modified Ashworth Scale (MAS)—evaluates the degree of spasticity by assessing resistance during passive joint mobilization. Scores range from 0 (no increase in muscle tone) to 4 (rigidity in flexion or extension). It is the most widely used clinical tool for spasticity assessment [17].
➢ Functional Independence Measure (FIM)—assesses the degree of functional independence in activities of daily living. It includes 18 items, divided into the motor domain (13 items: self-care, sphincter control, mobility, locomotion) and the cognitive domain (5 items: communication, social cognition). Each item is scored from 1 (total assistance) to 7 (complete independence) [18].
➢ EuroQol-5 Dimensions (EQ-5D)—a standardized questionnaire for evaluating health-related quality of life. It includes five domains: mobility, self-care, usual activities, pain/discomfort, and anxiety/depression, each rated on 3 or 5 severity levels. The questionnaire also incorporates a visual analogue scale (VAS) ranging from 0 (the worst imaginable health state) to 100 (the best imaginable health state) [19].

### 2.4. Therapeutic Intervention

All participants followed the standard individualized physical therapy program, which consisted of individual gym-based kinesiotherapy, occupational therapy, psychological counseling and at least one of the following therapeutic procedures: massage, electrotherapy; reflex bladder electrostimulation (TENS), pressotherapy lymph drainage, magnetotherapy, LASER therapy, thermotherapy, hydrokinetotherapy in the pool (thermal water 36 degrees for 20 min), The conservative kinesiotherapy program was identical for all four groups, comprising one session per day, five days per week, for a total of 10 sessions. Each session lasted 50 min and included, depending on the case, mobility and strengthening exercises, as well as exercises to improve motor control. All kinesiotherapy exercises were individualized according to each patient’s specific deficits, with frequency and intensity adjusted according to recorded progress and patients’ comorbidities. Occupational therapy focused on retraining transfers, activities of daily living (ADLs), and self-care skills. Within this rehabilitation routine, the main component of mobility training consisted of 10 sessions (2 weeks) of gait therapy using the Lokomat device, prescribed for patients in the L and VL groups. Patients in the VL and L groups underwent 10 sessions of robot-assisted gait training, each lasting 30 min. For Lokomat treatment, body weight support was initially set at 100% of each participant’s weight and was gradually reduced according to loading tolerance, but never below 25%. The treadmill speed was set at the most comfortable level for the patient. All treatment sessions were performed under the supervision of a specialized physiotherapist. Patients in the VL and V groups also received additional Vojta Therapy alongside the standard kinesiotherapy program. The Vojta therapy program was applied once daily for 10 consecutive days, with each session lasting approximately 30 min (Appendix A) [20,21]. Treatment was performed by a physiotherapist certified by the Vojta International Society.

### 2.5. Patients Enrolling

**Figure 1:**
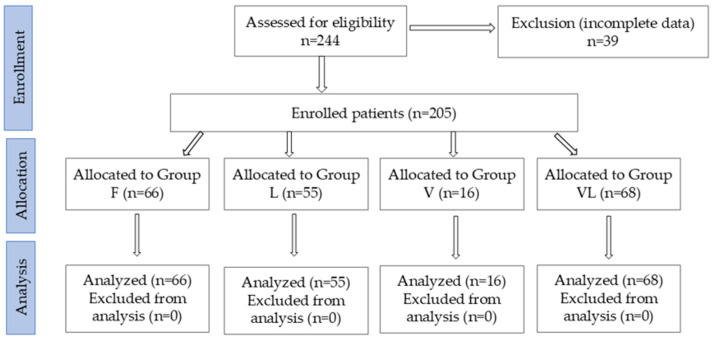
CONSORT flow diagram of the study.

Out of the 244 patients initially recruited, 205 met the inclusion criteria (Figure 1). Based on the initial admission assessment- considering the degree of neurological impairment (using the AIS), mobility status (using the EQ-5D scale), associated comorbidities, and complications related to spinal cord lesion—the treatment plan was established. Patients were subsequently assigned to four groups: Group F: patients who received only conventional rehabilitation therapy, consisting of individual gym-based kinesiotherapy, occupational therapy, psychological counseling, and at least one of the following therapeutic procedures: massage, electrotherapy, reflex bladder electrostimulation (TENS), pressotherapy lymph drainage, magnetotherapy, LASER therapy, thermotherapy, hydrokinesiotherapy in the pool. Group V: patients who underwent conventional rehabilitation therapy combined with Vojta Therapy. Group L: patients who underwent conventional rehabilitation therapy combined with Lokomat^®^ robotic gait training. Group VL: patients who underwent conventional rehabilitation therapy combined with Lokomat^®^ training and Vojta Therapy.

### 2.6. Statistical Analysis

All statistical analysis was generated using the JASP, (version 0.18.0) [Software]. Statistical significance was considered to be 0.05. The mean values of the determined parameters and the frequency of the intervals were compared. The tests used were Student’s *t*-test and Mann–Whitney U test, depending on the homogeneity/heterogeneity of the dispersion between the two determinations at the cohort level. Normality testing was performed using the Shapiro–Wilk test. To evaluate the differences between the mean values of the measurements of the four study groups, we used the ANOVA method. The Levene test was used to test the homogeneity of variances. The Kruskal–Wallis nonparametric analysis was used in the case of unequal variances. The multiple linear regression model was used to identify predictive factors for the FIM independence score.

## 3. Results

Analysing the data from Table 2, we observe that the mean age in the VL group was the lowest, followed by the V, L, and F groups, respectively. Age variation was generally similar across groups, with the V group showing the highest variability (SD). In the F and L groups, the distribution was skewed toward higher values, suggesting a greater proportion of older patients (skewness test). Out of the total 205 patients, 65.36% were male. The majority of participants were recruited from urban areas. From an etiological perspective, more than 60% of the cases included in the study were due to traumatic spinal cord injuries.

The distribution of patients according to the AIS varied across groups. Proportionally, the F group had a large percentage of patients with grade C/D (motor-incomplete), with grade D representing 32/66 (~48.5%).

The VL group showed approximately 44% of patients with grade C and D (30/68). The V group, although smaller (N = 16), had a high proportion of grade D cases (50%). Regarding spasticity, the VL group had significantly higher proportions of scores 2 and 3 (moderate to severe spasticity) compared to the other groups, whereas the V group showed higher frequencies at scores 1–2. Overall, spasticity intensity appeared more pronounced in the VL group and moderate in the F and L groups. In Group F, 23 patients with paraparesis (34.85%) and 43 patients with tetraparesis (65.15%) were included. In Group V, 6 patients with paraparesis (37.50%) and 10 patients with tetraparesis (62.50%) were assigned. In the L and VL groups, patients with paraparesis predominated, accounting for approximately 80% in each group.

Regarding the ASIA motor and sensitive subscores, Group F had the highest mean motor score (~74.6 points), followed by groups L and V (~64–65 points). The mean value in the VL group was the lowest (~57.4). Variability was greater in the V group (SD = 22.55) compared to the other groups (Table 2).

### 3.1. Neurological Level of Injury

Analysis of the dataset presented in Table 3 suggests that the most frequent levels of injury were C7 (42 patients, approximately 21% of the total 205 recruited patients) and L1 (40 patients, approximately 20%), followed by L2 (12.68%) and T10 (8.78%)**.** All *p*-values (chi-square test) were >0.05; therefore, there was insufficient evidence to reject the null hypothesis of independence, indicating no statistically significant association between lesion level and study group.

### 3.2. Mobility Score Determined with EQ-5D

**Figure 2:**
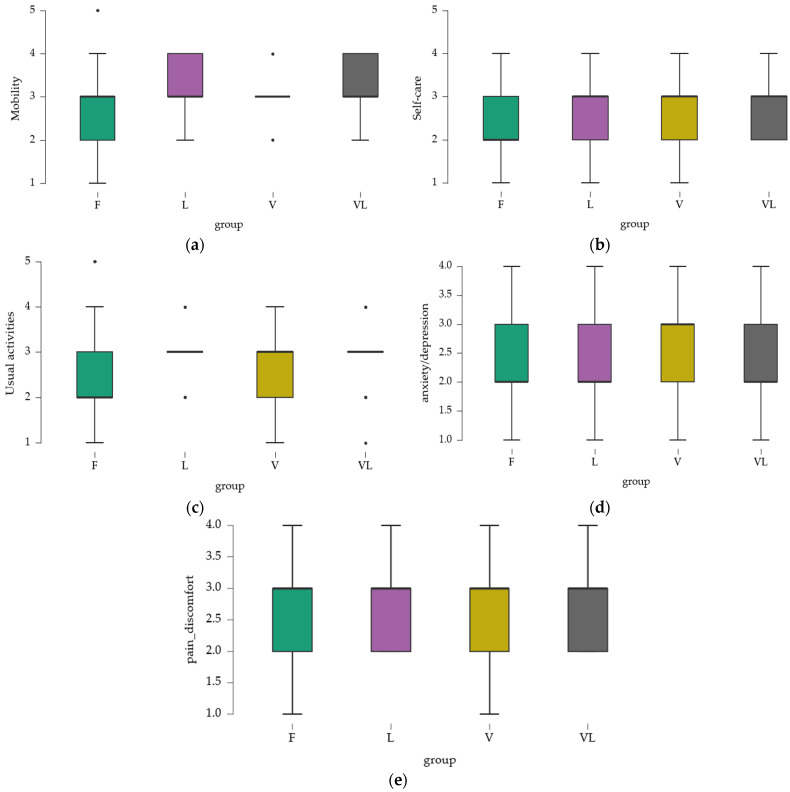
Mobility score assessed with EQ-5D. (**a**) mobility; (**b**) self-care; (**c**) usual activities; (**d**) pain/discomfort; (**e**) anxiety/depression.

The results obtained for the EQ-5D evaluation domains provide insight into the perceived health status across different aspects of quality of life. Mean values were calculated separately for each domain (Figure 2).

#### 3.2.1. Mobility

Mean values ranged from 2.742 (Group F) to 3.191 (Group VL), indicating that, on average, participants experienced mobility limitations, particularly in the VL group. Minimum and maximum values revealed the presence of individuals with nearly normal mobility (score 1) and others with more severe limitations (score 5). However, the majority of participants reported some degree of mobility difficulty, with varying levels of severity.

#### 3.2.2. Self-Care

Mean values were closer across groups, with the highest observed in the VL group (2.824), indicating deficits in self-care. The range of minimum and maximum values suggested that while some individuals experienced only minor difficulties, others presented with more significant challenges.

#### 3.2.3. Usual Activities

Mean values indicated that, overall, participants perceived moderate difficulties in performing usual activities, with a slight increase in the VL group. Variations suggested experiences ranging from almost normal (minimum 1) to significant difficulties (maximum 5).

#### 3.2.4. Pain and Discomfort

Mean values were around 2.6–2.7, reflecting a moderate level of discomfort. Variability indicated that while some participants reported mild pain, others experienced more severe discomfort.

#### 3.2.5. Anxiety and Depression

Mean values ranged from 2.2 to 2.5, suggesting a moderate level of perceived psychological problems. The V group had a slightly higher mean, which may indicate a greater perception of anxiety and depression.

### 3.3. Recovery Treatment

All patients underwent conventional kinesiotherapy (100%). A total of 84 (40.97%) patients received Vojta Therapy, and 123 (60%) patients underwent Lokomat training (Table 4). Within the types of therapeutic interventions, the most frequently recommended procedures besides the standard neurorehabilitation therapy, were: sedative massage (181 patients) and electrotherapy (166 patients), followed by TENS for neurogenic bladder (72 patients) and psychological counseling (69 patients). The least commonly used were thermotherapy (25 patients) and magnetic therapy (34 patients), while LASER therapy showed a moderate frequency (41 patients).

### 3.4. Spasticity Progression at the Cohort Level

**Figure 3:**
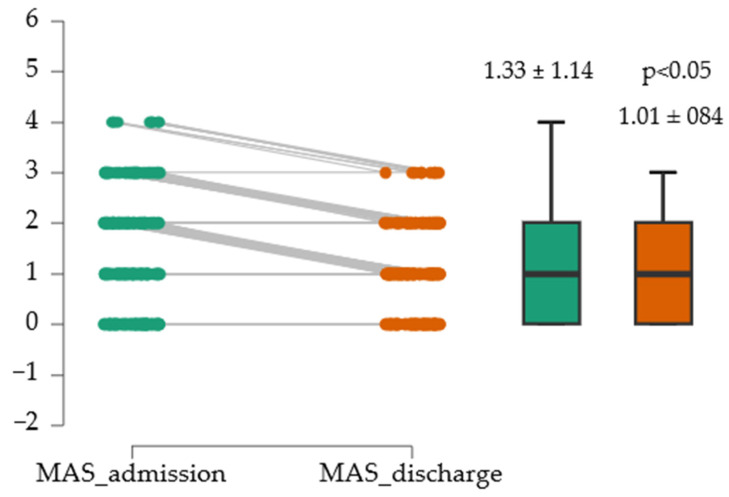
Evolution of MAS medium score.

For the assessment of spasticity at admission and discharge, the Modified Ashworth Scale (MAS) was used. To test the normality of the distribution, the Shapiro–Wilk Test of Normality was applied. Given the non-normal distribution of the MAS score at discharge (W = 0.589, *p* < 0.05), the Wilcoxon signed-rank test was used to compare the results. Figure 3 suggests that the difference was statistically significant, with a modest effect size (0.014).

### 3.5. Differences in Spasticity Across Study Groups

**Figure 4:**
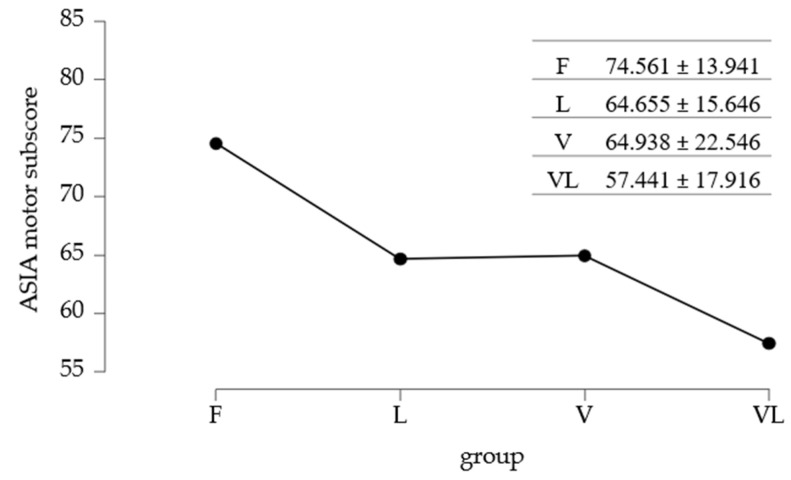
Mean values of ASIA motor subscore/study group.

The mean values of the ASIA motor subscore are summarized in Figure 4.

To identify differences between the mean ASIA motor subscores, the standard Post Hoc test was applied. Data analysis from Table 5 showed significant differences between Group F and Group L, as well as between Group F and Group VL.

### 3.6. Evolution of Functional Independence at the Cohort Level

**Figure 5:**
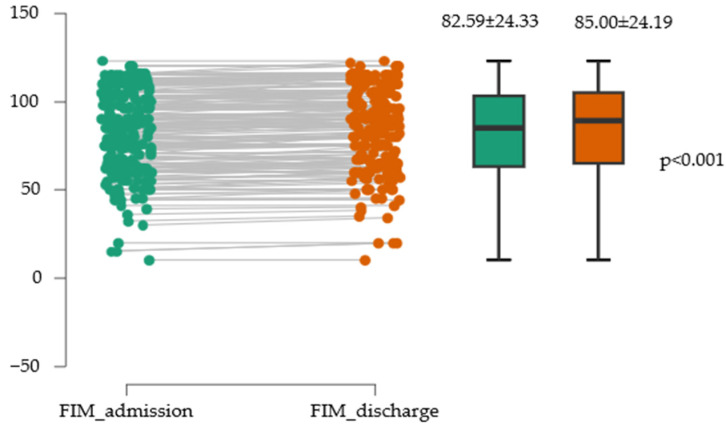
Evolution of functional independence assessed with the FIM scale.

The two variables (FIM_admission and FIM_discharge) showed very similar means, with relatively comparable dispersion and variability, indicating that the scores for the two scales were consistent in terms of central tendency and dispersion within this sample (Figure 5).

There was a statistically significant difference between the two measurements, with FIM_admission having a mean approximately 2.4 points lower than FIM_discharge. This difference is considerable from a practical standpoint, representing a large effect. The effect size value of −0.845 indicates a large to very large effect, suggesting that the difference between the two measures has substantial practical relevance (Table 6).

### 3.7. Differences in Functional Independence Between Study Groups

**Figure 6:**
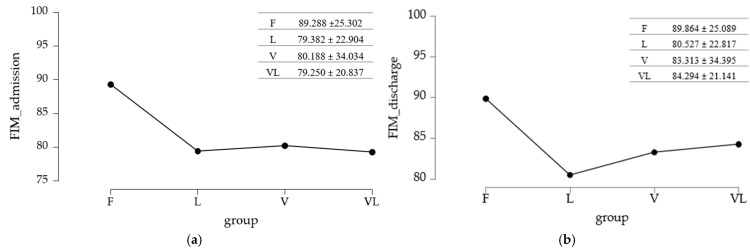
Differences in functional independence between study groups: (**a**) initial assessment; (**b**) final assessment.

The mean values of functional independence across study groups are summarized in Figure 6.

#### 3.7.1. Baseline Assessment

To evaluate the differences across study groups, the equality of variances was tested using Levene’s test.

Since *p* < 0.05 and the variances were not equal between groups, a nonparametric analysis was applied (Kruskal–Wallis test followed by a post hoc test that accounts for unequal variability—Dunn’s test with Holm or Bonferroni adjustments) (Table 7). The Kruskal–Wallis test (*p* = 0.017) indicated that at least one group differed in the distribution of the measured variable, but did not specify which groups differed from each other. To identify the differing pairs, a Dunn’s post hoc test was performed.

#### 3.7.2. Final Assessment

The methodology used for the baseline evaluation was maintained, given the inequality of variances identified at the reassessment of the FIM score. The *p* < 0.05 value obtained with Levene’s test led to the application of the Kruskal–Wallis test and Dunn’s Post Hoc test.

The Kruskal–Wallis test analysis suggested that there were no significant differences between the compared group pairs (*p* = 0.077), a result further supported by Dunn’s Post Hoc test (with Holm/Bonferroni adjustments). The results are summarized in Table 8.

### 3.8. Predictive Factors for the FIM Independence Score

A linear regression model was applied to identify potential predictors for the FIM score (Table 9). The regression model explained approximately 47% of the variance in the FIM scale (R^2^ = 0.470).

The global ANOVA test of the model (Table 10) indicated that the overall model was significant, meaning that at least one of the predictors had a significant effect on the FIM scale (F = 21.742, *p* = 1.65 × 10^−23^).

The adjusted intercept (H_1_) was 22.372 (*p* = 0.007)**,** statistically significant (Table 11). The coefficient of 0.760 (*p* = 1.32 × 10^−5^) indicated that for each additional point in the ASIA motor score, the FIM scale increased by approximately 0.76 points, suggesting a positive and significant relationship. The standardized coefficient (0.560) reflected a moderate influence of this covariate. Data analysis suggested that only the VL group had a significant coefficient (10.341, *p* = 0.004), meaning that patients in this group had, on average, FIM scores almost 10.3 points higher compared with the reference group. The coefficient of 0.035 (*p* = 0.050) indicated a tendency for FIM scores to increase with a longer time since disease onset, although the effect was marginally significant. Therefore, the most relevant predictor of the FIM score was the ASIA motor score, while group membership (particularly the VL group) also had a significant effect.

## 4. Discussion

The present study aimed to analyze the combined effect of Vojta Therapy and robot-assisted gait training (Lokomat^®^) on motor function recovery in patients with spinal cord lesion (SCL), based on the hypothesis that this synergy could lead to superior outcomes compared with standard rehabilitation programs. Four rehabilitation strategies were analyzed and compared in SCL patients: conventional rehabilitation, conventional rehabilitation combined with Vojta Therapy, conventional rehabilitation combined with Lokomat training, and an integrative protocol including both Vojta and Lokomat therapies alongside conventional therapy.

Review of the literature shows that Lokomat training can improve walking ability, muscle strength, and activities of daily living, particularly in the subacute phase and with longer training durations (>2 months), suggesting a dose- and time-dependent window for neuroplastic responses [22]. The benefits of Lokomat in increasing walking distance, speed, and mobility are also supported by the systematic review published by A.R. Alashram (2021) [5].

The groups differed in demographic and severity profiles: groups F and VL had a higher proportion of men, and AIS categories B/C/D were unevenly distributed across groups. The VL group included younger patients, with a greater proportion of spasticity (Ashworth 2–3) and a different AIS distribution compared with the other groups. The min-max ranges indicated wide age variability.

Our results highlighted that patients who received combined Lokomat and Vojta therapy (VL group) achieved significant improvements in functional independence, compared with those in the L group (Lokomat + conventional physiotherapy). The statistically significant difference observed in FIM discharge scores, which remained after adjustments for multiplicity, suggests that adding Vojta Therapy to robotic gait training has an additive effect on functional recovery. In real-world rehabilitation terms, a 2.4-point FIM increase indicates a small but measurable functional improvement. It might correspond to a patient needing slightly less physical help for dressing or transfers, or performing one ADL component more independently, or that his mobility in bed transfers has increased. In a retrospective cohort study entitled “Association between the Functional Independence Measure following spinal cord injury and long-term outcomes”, Cohen’s study results demonstrate that even small improvements in FIM at discharge translate into meaningful long-term functional, social, and economic benefits. Thus, a gain of even 2–3 FIM points can have a measurable real-world impact on independence and care costs.

This evidence reinforces the clinical relevance of FIM changes as predictors of rehabilitation effectiveness and long-term autonomy in SCI populations [23].

The study by Mónica Alcobendas-Maestro et al. (2012), including 80 patients with incomplete SCI, similarly suggested that robot-assisted therapy may provide additional benefits in walking recovery (6-min walk test), improving patient independence (locomotor section of FIM) and reducing reliance on walking aids [7]. Although patients in the VL group had lower ASIA motor and sensory scores at admission compared with the L group, they achieved higher functional independence at discharge. This supports the hypothesis that combined therapy stimulates neuroplastic and functional reorganization mechanisms even in patients with more severe neurological impairment [24]. While the literature on Vojta combined with robotic rehabilitation is limited, evidence suggests that Vojta Therapy activates reflex locomotor patterns, which may be integrated into functional motor schemes. Thus, the combination of the two methods may offer additional advantages by facilitating motor control and coordination [25]. The combined use of Vojta therapy and Lokomat training appears to enhance motor recovery in spinal cord injury (SCI) through synergistic neuroplastic mechanisms. Vojta therapy activates innate motor pathways via reflex stimulation of proprioceptive and tactile receptors, eliciting coordinated global motor responses even in patients with severe neurological impairment. This so-called “activation” may “prime” the central nervous system, increasing excitability within residual corticospinal tracts and central pattern generators (CPGs) in the lumbosacral cord. This boosts corticoreticulo–spinal excitability and postural set; in addition, Lokomat gait training provides high-repetition, task-specific stepping with precise kinematics and load management (BWS, Guidance), the core ingredients for activity-dependent plasticity. Together, Vojta sets the system to “ready to learn”, while Lokomat supplies the intensive, specific practice the CNS needs to consolidate change. The resulting bidirectional neuromodulation (bottom-up proprioceptive activation and top-down voluntary drive) likely underpins the superior outcomes observed in the combined group. Similar synergistic neuroplastic effects have been reported in studies combining sensory stimulation or FES with robotic gait training [26].

A pilot study by J-del-Ama et al. demonstrated that integrating reflex-based motor activation with robotic training is both feasible and safe, reinforcing the hypothesis that combined interventions may provide further benefits [27].

Regarding spasticity, our comparative analysis of MAS scores at discharge revealed important differences across groups. In the F group (conventional therapy), nearly half of patients scored MAS 0–1, suggesting moderate reduction in spasticity, though ~27% still scored ≥2. This aligns with the literature indicating that classical rehabilitation can reduce spasticity, but effects are often limited and variable [28]. In the V group (Vojta Therapy), distribution was more balanced, with a higher proportion of patients scoring 2–3, possibly reflecting both the intensity of reflex stimulation and limitations of this method in reducing severe spasticity. Recent studies indicate that Vojta may influence neuromuscular activation and postural control, but direct evidence for its impact on spasticity remains scarce [9].

The L group (Lokomat^®^) showed a relatively high proportion of patients scoring MAS = 2 (50%), suggesting that although robotic gait training contributes to motor function and endurance, its impact on spasticity is less pronounced. This is consistent with meta-analyses reporting modest or inconsistent effects of RAGT on spasticity, with significant benefits seen only in certain patients, particularly in the subacute phase [5,29].

Interestingly, the VL group (Vojta + Lokomat) showed heterogeneity: while 26.5% of patients scored 0 (no spasticity), the group also had the highest proportion with a score of 4 (10.3%). This suggests that combined intervention may be highly effective for some, but in others spasticity persisted or even worsened. A possible explanation lies in individual variability in response to multimodal therapy, influenced by lesion severity (AIS A-D), neurological level, and time since injury [30]. Overall, our data confirm that spasticity outcomes following rehabilitation vary across groups; conventional therapy and Vojta showed trends toward reducing severe cases (score 4), whereas Lokomat mainly provided standardized movement patterns without consistent spasticity reduction. Clinically, this emphasizes the need for individualized and multimodal strategies. While spasticity can sometimes support postural tone, it must be monitored and managed to avoid interference with functional recovery [31].

Analysis of therapeutic interventions showed that conventional procedures—especially sedative massage and electrotherapy—were the most frequently applied across groups. This reflects current clinical practice in spinal rehabilitation, where passive and adjunctive therapies are used for pain control, spasticity reduction, and mobility facilitation [32]. However, the literature shows that while these methods may alleviate symptoms, their long-term effect on motor recovery is limited, requiring integration with active, task-specific programs [33].

Modern interventions showed that Lokomat was used in over half the cohort, while Vojta Therapy was applied to fewer patients, primarily concentrated in the VL group. These findings confirm the global trend toward integrating RAGT in SCI rehabilitation protocols, particularly in subacute and chronic patients, where RAGT has shown walking improvements [7,34].

The VL group illustrates the concept of multimodal intervention, combining reflex-based motor activation (Vojta) with the intensity and repetitiveness of RAGT. Despite its potential, our analysis showed that VL patients often presented comorbidities (e.g., neurogenic bladder), which may explain slower motor progress. Nevertheless, VL patients achieved significantly greater functional independence improvements compared with the L group. This is consistent with literature showing that initial lesion severity and comorbidities directly influence rehabilitation outcomes regardless of therapy [35]. Thus, our data confirm that classical rehabilitation remains essential, but insufficient to achieve maximal independence and QoL. Introducing modern technologies such as Lokomat and integrating reflex-based therapies such as Vojta may provide additional benefits, particularly in intensive, long-term programs. Clinically, this emphasizes the importance of multimodal strategies tailored by ASIA classification, AIS grade, neurological level, and injury stage.

### Strengths and Limitations of the Study

The strengths of this study lie in the fact that, to our knowledge, it is the first study conducted in Romania to comparatively investigate the benefits of Vojta Therapy in this pathology. Another strength is the inclusion of a substantial number of patients, which adds weight to the analysis. 

At the same time, the limitations of this study must be acknowledged to properly contextualize the findings. First, the lack of randomization may influence the comparative validity of the study groups and introduce potential confounding factors. Second, the younger age of patients in groups V and VL may affect the generalizability of the results, since neuroplastic potential tends to decline with age, influencing the capacity for neural reorganization and motor recovery.

Furthermore, the absence of long-term follow-up restricts the evaluation of the durability of therapeutic effects, particularly regarding the transfer of motor gains to activities of daily living (ADL). Another methodological limitation is the lack of stratification by lesion level and disease duration, as both variables significantly influence neuroplastic processes, treatment responsiveness, and functional prognosis. 

## 5. Conclusions

This study demonstrates that integrating Vojta therapy with robotic gait training can yield greater improvements in motor recovery and functional independence than conventional or single-modality rehabilitation. The combined intervention likely enhances neuroplastic activation through simultaneous proprioceptive stimulation and task-specific locomotor training. Patient outcomes are strongly influenced by the ASIA classification, AIS grade, and neurological level of injury, underscoring the need for individualized, multimodal rehabilitation programs. Our findings support the need for prospective, multicenter, randomized studies with long-term follow-up to confirm these observations and to identify subgroups of patients who may benefit most from this combined therapeutic strategy. Future prospective randomized controlled trials with standardized protocols, long-term follow-up, and neurophysiological outcome measures are warranted to confirm these findings and clarify the mechanisms underlying synergistic neuroplasticity in SCL rehabilitation.

## Figures and Tables

**Table 1:** AIS (ASIA Impairment Scale)—classification of spinal cord injury.

AIS Grade	Description
A	Complete injury- no motor or sensory function below the level of injury.
B	Incomplete injury- sensory function is preserved below the level of injury, but no motor function is present.
C	Incomplete injury—motor function is preserved below the level of injury, but the majority of key muscles have a strength grade < 3/5.
D	Incomplete injury—motor function is preserved below the level of injury, and the majority of key muscles have a strength grade ≥ 3/5.
E	Normal motor and sensory function.

**Table 2:** Baseline characteristics.

Parameter	F	L	V	VL	Total Group
Patients, N	66	55	16	68	205
Age, M, SD (years)	59.03 ± 14.36	50.16 ± 14.35	45.93 ± 16.73	45.11 ± 13.91	51.01 ± 15.46
Male, N	40	35	10	49	134
Rural area, N	22	26	3	31	82
Traumatic etiology, N (%)	23	44	6	54	127
Onset, M, SD (months)	88.79 ± 77.63	104.64 ± 90.68	64.12 ± 53.67	59.05 ± 48.15	81.25 ± 73.65
Tetraparesis, N (%)	43 (65.15)	11 (20)	10 (62.50)	14 (20.59)	
Paraparesis, N(%)	23 (34.85)	44 (80)	6 (37.50)	54 (79.41)	
AIS					
Grade A, N	5	8	2	17	32
Grade B, N	6	17	5	21	49
Grade C, N	23	16	1	19	59
Grade D, N	32	14	8	11	65
ASIA motor subscore					
M ± SD	74.56 ± 13.94	64.65 ± 15.65	64.94 ± 22.55	57.44 ± 17.92	65.47 ± 17.83
Min–Max	40.00–97.00	35.00–92.00	5.00–90.00	18.00–97.00	5.00–97.00
ASIA sensory subscore					
M ± SD	87.88 ± 17.77	77.51 ± 19.01	77.13 ± 29.19	70.29 ± 22.84	78.42 ± 21.94
Min–Max	44.00–110.00	36.00–106.00	14.00–110.00	20.00–110.00	20.00–110.00
Modified Ashworth Scale					
Score 0, N (%)	28	13	3	18	62
Score 1, N (%)	20	19	3	12	54
Score 2, N (%)	13	14	8	21	56
Score 3, N (%)	5	8	2	10	25
Score 4, N (%)	0	1	0	7	8

N—number of participants; M—mean (average); SD—standard deviation; F—conventional physiotherapy group; L—Lokomat group; V—Vojta group; VL—Vojta + Lokomat group, ASIA—American Spinal Injury Association.

**Table 3:** Neurological level of injury determined for each patient.

Injury Level	Group				Total, N (%)
	F, N (%)	L, N (%)	V, N (%)	VL, N (%)	
C4	1.00 (1.51)	0.00 (0.00)	0.00 (0.00)	0.00 (0.00)	1.00 (0.49)
C5	1.00 (1.51)	0.00 (0.00)	0.00 (0.00)	5.00 (7.35)	6.00 (2.97)
C6	0.00 (0.00)	2.00 (3.64)	0.00 (0.00)	2.00 (2.94)	4.00 (1.95)
C7	18.00 (27.27)	6.00 (10.90)	4.00 (25.00)	14.00 (20.58)	42.00 (20.49)
C8	2.00 (3.03)	6.00 (10.90)	2.00 (12.50)	4.00 (5.88)	14.00 (6.83)
T1	0.00 (0.00)	0.00 (0.00)	0.00 (0.00)	2.00 (2.94)	2.00 (0.98)
T2	1.00 (1.51)	0.00 (0.00)	0.00 (0.00)	0.00 (0.00)	1.00 (049)
T4	0.00 (0.00)	1.00 (1.81)	0.00 (0.00)	1.00 (1.47)	2.00 (0.98)
T5	0.00 (0.00)	2.00 (3.64)	0.00 (0.00)	0.00 (0.00)	2.00 (0.98)
T6	0.00 (0.00)	2.00 (3.64)	0.00 (0.00)	5.00 (7.35)	7.00 (3.41)
T7	0.00 (0.00)	1.00 (1.81)	0.00 (0.00)	2.00 (2.94)	3.00 (1.46)
T8	0.00 (0.00)	1.00 (1.81)	1.00 (6.25)	0.00 (0.00)	2.00 (0.98)
T9	0.00 (0.00)	0.00 (0.00)	1.00 (6.25)	1.00 (1.47)	2.00 (0.98)
T10	7.00 (10.60)	5.00 (9.09)	1.00 (6.25)	5.00 (7.35)	18.00 (8.78)
T11	0.00 (0.00)	0.00 (0.00)	0.00 (0.00)	1.00 (1.48)	1.00 (049)
T12	2.00 (3.03)	2.00 (3.64)	0.00 (0.00)	1.00 (1.47)	5.00 (2.44)
L1	11.00 (16.67)	13.00 (23.64)	2.00 (12.50)	14.00 (20.59)	40.00 (19.51)
L2	11.00 (16.67)	7.00 (12.73)	2.00 (12.50)	6.00 (8.82)	26.00 (12.68)
L3	4.00 (6.06)	3.00 (5.45)	1.00 (6.25)	2.00 (2.94)	10.00 (4.88)
L4	7.00 (10.60)	4.00 (7.27)	2.00 (12.50)	3.00 (4.41)	16.00 (7.80)
L5	1.00 (1.51)	0.00 (0.00)	0.00 (0.00)	0.00 (0.00)	1.00 (0.49)

C—cervical; T—thoracic; L—lumbar; N—number of participants; (**%**)—percentages.

**Table 4:** Distribution of patients according to frequency of therapeutic interventions.

Therapy	Group				Total
	F, N	L, N	V, N	VL, N	
Vojta	0	0	16	68	84
Lokomat	0	55	0	68	123
PC	15	14	8	32	69
MDF	12	7	5	10	34
LASER	19	9	1	12	41
ET	58	44	11	53	166
TENS	14	23	5	30	72
Massage	60	50	14	57	181
TT	8	6	3	8	25
PLD	21	25	3	33	82

F—conventional physiotherapy group; L—Lokomat group; V—Vojta group; VL-Vojta + Lokomat group. PC—psychological counseling; MDF—magnetic therapy; LASER—light amplification by stimulated emission of radiation; ET—electrotherapy; TENS—transcutaneous electrical nervous stimulation; TT—thermotherapy; PLD—pressotherapy lymph drainage.

**Table 5:** Post Hoc Comparisons-group.

		Mean Difference	SE	t	*p*_bonf_	*p*_holm_
F	L	9.906	3.020	3.281	0.007	0.006
	V	9.623	4.609	2.088	0.228	0.114
	VL	17.119	2.858	5.990	5.710 × 10^−8^	5.710 × 10^−8^
L	V	−0.283	4.698	−0.060	1.000	0.952
	VL	7.213	2.999	2.405	0.102	0.068
V	VL	7.496	4.595	1.631	0.626	0.209

L—Lokomat group; V—Vojta group; SE—Standard error; t—test value; *p*_bonf_—*p*-value adjusted by Bonferroni correction; *p*_holm_—*p*-value adjusted by Holm correction.

**Table 6:** Paired *t*-test for FIM_ADM/FIM_DIS.

Measure 1	Measure 2	t	df	*p*	MD	SE Dif.	Cohen’s d	SE Cohen’s d
FIM_ADM	FIM_DIS	−12.097	204	<0.001	−2.410	0.199	−0.845	0.010

FIM_ADM—Functional Independence Measure score at admission (initial evaluation); FIM_DIS-Functional Independence Measure score at discharge (final evaluation); t—t-statistic; df—degrees of freedom; *p*—significance level; MD—mean difference; SE Dif.—standard error of the difference; Cohen’s d—standardized effect size; SE Cohen’s d—standard error of Cohen’s d.

**Table 7:** Dunn’s Post Hoc Comparisons-group.

Comparison	z	W_i_	W_j_	*p*	*p*_bonf_	*p*_holm_
F-L	2.648	121.477	92.818	0.008	0.049	0.041
F-V	0.916	121.477	106.344	0.360	1.000	1.000
F-VL	2.827	121.477	92.515	0.005	0.028	0.028
L-V	−0.803	92.818	106.344	0.422	1.000	1.000
L-VL	0.028	92.818	92.515	0.977	1.000	1.000
V-VL	0.839	106.344	92.515	0.401	1.000	1.000

z—z-statistic for the pairwise comparison Wi, Wj—mean ranks of the two groups compared; *p*—unadjusted *p*-value; *p*_bonf—_*p*-value adjusted for multiple comparisons (Bonferroni correction); *p*_holm_—*p*-value adjusted for multiple comparisons (Holm correction).

**Table 8:** Dunn’s Post Hoc Comparisons-group.

Comparison	z	W_i_	W_j_	*p*	*p*_bonf_	*p*_holm_
F-L	2.532	116.826	89.427	**0.011**	0.068	0.068
F-V	0.542	116.826	107.875	0.588	1.000	1.000
F-VL	1.700	116.826	99.412	0.089	0.534	0.445
L-V	−1.096	89.427	107.875	0.273	1.000	1.000
L-VL	−0.929	89.427	99.412	0.353	1.000	1.000
V-VL	0.514	107.875	99.412	0.607	1.000	1.000

z—z-statistic for the pairwise comparison Wi, Wj—mean ranks of the two groups compared; *p*- unadjusted *p*-value; *p*_bonf_—*p*-value adjusted for multiple comparisons (Bonferroni correction); *p*_holm_—*p*-value adjusted for multiple comparisons (Holm correction).

**Table 9:** Linear regression model for identifying predictive factors for FIM_discharge.

Model	R	R^2^	Adjusted R^2^	RMSE
H_0_	0.000	0.000	0.000	24.199
H_1_	0.686	0.470	0.449	17.970

Model—tested regression model (H_0_—null model, H_1_—fitted model with predictors); R—multiple correlation coefficient; R^2^—coefficient of determination (proportion of variance explained by the model); Adjusted R^2^—coefficient of determination adjusted for the number of predictors; RMSE—root mean square error (measure of prediction error).

**Table 10:** Statistics of the regression model for the independence score FIM.

Model		Sum of Squares	df	Mean Square	F	*p*
H_1_	Regression	56,168.119	8	7021.015	21.742	1.651 × 10^−23^
	Residual	63,291.881	196	322.918		
	Total	119,460.000	204			

Model—regression model tested (H_1_—fitted model with predictors); Sum of Squares—total variance explained by the model (regression) or left unexplained (residual); df—degrees of freedom; Mean Square—average variance (Sum of Squares divided by df); F—F-statistic testing overall model significance; *p*—significance level.

**Table 11:** Statistically significant predictors of the independence score FIM.

Model		Unstandardized	Standard Error	Standardized ^a^	t	*p*
H_0_	(Intercept)	85.000	1.690		50.292	6.361 × 10^−117^
H_1_	(Intercept)	22.372	8.234		2.717	0.007
	ASIA motor subscore	0.760	0.170	0.560	4.470	1.322 × 10^−5^
	group (L)	−1.191	3.451		−0.345	0.730
	group (V)	2.257	5.188		0.435	0.664
	group (VL)	10.341	3.562		2.903	0.004
	Onset(months)	0.035	0.018	0.107	1.969	0.050
	MAS_discharge	−1.132	1.632	−0.039	−0.694	0.489
	Age	−0.128	0.090	−0.082	−1.418	0.158
	ASIA sensory subscore	0.183	0.135	0.166	1.360	0.175

Unstandardized—raw regression coefficient (B); Standard Error—standard error of the coefficient; Standardized ^a^—standardized beta coefficient (relative importance of the predictor, when applicable); t—t-statistic testing whether the coefficient differs from zero; *p*—significance level; MAS_discharge- Modified Ashworth Scale at discharge.

## Data Availability

The data presented in this study are not publicly available due to privacy and ethical restrictions, as they are archived in the hospital’s institutional database.

## Appendix A. The Vojta Therapy Program

The intervention was based on reflex locomotion, incorporating two main coordination techniques- reflex rolling and reflex crawling. Each technique began with a one-minute resting period, followed by manual stimulation of specific trigger zones using firm, sustained, and painless pressure. After each stimulation, the patient remained in the same position for one additional minute to allow the reflex responses to persist [19].

Reflex rolling-supine position: the patient was placed in supine with eyes open and the head rotated approximately 30° toward the side of stimulation. After the initial resting period, the physiotherapist applied pressure in the thoracic region, between the 7th and 8th ribs. Reflex rolling-side-lying position: the patient was placed in side-lying. Stimulation was applied at the medial border of the scapula and the anterior superior iliac spine. Reflex crawling-prone position: the patient was placed in prone and received continuous stimulation for reflex locomotion at the following points: medial humeral epicondyle, acromion, thoracic region, medial border of the scapula, iliac spine, medial femoral epicondyle, calcaneus, and gluteal region [20].
